# Bernard Soulier syndrome: A case report from Pakistan

**DOI:** 10.1002/ccr3.7767

**Published:** 2023-07-30

**Authors:** Iqra Effendi, Ahsan Nadeem, Sara Sarfraz, Mubasshar Shahid, Minaam Farooq, Ayush Anand

**Affiliations:** ^1^ CMH Lahore Medical College and Institute of Dentistry Lahore Pakistan; ^2^ Rashid Latif Medical College Lahore Pakistan; ^3^ Mayo Hospital Lahore Pakistan; ^4^ BP Koirala Institute of Health Sciences Dharan Nepal

**Keywords:** Bernard soulier syndrome, case report, hereditary, platelet disorder

## Abstract

Bernard Soulier Syndrome should be suspected in patients with bleeding disorder symptoms and significant family history, where consanguineous marriages are common. Diagnosis can be confirmed using a ristocetin test and a peripheral blood smear.

## 1 INTRODUCTION

Bernard Soulier syndrome (BSS) is a rare inherited platelet disorder with a prevalence of one in a million people.^1^ It is caused by genetic abnormalities in the function of the GPIb‐V‐IX complex, which is required to bind von‐Willebrand factor IX.^2^ The defective GPIb‐V‐IX complements cannot attach to the endothelium, leading to adhesion failure, and increased patient bleeding tendencies. Mutations in four genes (i.e., GPIbα, GPIbβ, GPIX, and GPV) are mainly responsible for creating the products that combine to form the defective complex.^3^ These mutations have been documented as missense, frameshift, or deletions.^4^ Defects in any of these genes can hinder the ability of platelets to function normally. Although most cases of BSS are acquired as an autosomal recessive hereditary trait; some autosomal dominant inheritance for missense mutations have also been noted.^5^

The clinical signs of BSS involve mucocutaneous bleeding, with the most common presentations being unconstrained bruising, gum bleeding, and epistaxis.^6^ In addition to the typical clinical signs, the disorder can also present with menorrhagia, gastrointestinal bleeding, and profuse bleeding after surgery or injury that may be troublesome to control.^6^ These clinical signs of BSS and the associated thrombocytopenia are more likely to be noted in early childhood. Still, it is often misdiagnosed as other bleeding disorders, such as immune thrombocytopenia (ITP).^7^ However, in BSS, giant platelets without platelet aggregation, when reacted with ristocetin in flow cytometry, are specific to the disease. Due to the rare presentation of BSS and its similar symptomatology to other bleeding disorders, cases can often go undiagnosed or misdiagnosed. In developing countries, where healthcare resources are limited, diagnosing BSS correctly and utilizing resources for appropriate treatment is paramount.^8^ Our aim in this case report is to present the case of a nine‐year‐old girl diagnosed with BSS, to evaluate how the disease was managed, and to identify any shortcomings in its treatment.

## 2 CASE PRESENTATION

A nine‐year‐old Asian girl presented to the outpatient department complaining of bleeding from one of her primary upper molar teeth for the past 2 days. The bleeding persisted despite the parents' attempted home treatments with tranexamic acid–soaked gauze. Upon general physical examination, the patient was alert, oriented, and cooperative. The rest of the systemic examination was unremarkable. Initial laboratory investigations showed anemia and thrombocytopenia (platelet count of 60 × 10^9^/L). Following this, we admitted the patient to the pediatric ward, where she was given 500 mg tranexamic acid injections thrice daily and 345 mL of platelet transfusion after crossmatching. This treatment successfully resolved the patient's bleeding within a day, and the patient was discharged.

The patient was a known case of BSS diagnosed at 8 months of age. Her first episode that perpetuated the need for further investigation was in 2013, at 6 months of age, when she had unstoppable bleeding from both thighs during her routine six‐month vaccination. Around this time, she also started to develop multiple tender bruises on her arms and legs, with no history of trauma, which persisted for about 2 months. The patient's personal history showed that her parent's marriage was consanguineous, with both parents having no history of bleeding disorders. She was the oldest of three siblings, and her four‐year‐old brother was also diagnosed with BSS shortly due to persistent mosquito‐bite marks and bruises. The brother's circumcision was delayed due to the diagnosis. The parents stated that episodes have been less frequent and severe than the sister's, with a faster recovery time. Upon questioning both children's growth and developmental parameters, the parents stated that both achieved developmental milestones as expected and performed well in school. Apart from the bleeding episodes, they said their children could participate in their daily activities with their family and peers. Along with the current management, bone marrow transplantation was an option they had been informed of but could not receive at present due to financial limitations.

For further investigation, hematological tests were conducted at the National Institute of Blood Disease and Bone Marrow Transplantation in Lahore. The lab investigations revealed anemia (hemoglobin 9.5 g/dL) and thrombocytopenia (95 × 10^9^/L). A peripheral blood smear revealed hypochromic microcytic red blood cells, anisocytosis, and large platelets. The bleeding time of the patient was high, whereas prothrombin time, activated partial thromboplastin time, and international normalized ratio were within normal ranges. Platelet aggregation with ristocetin was carried out, which revealed failed aggregation. The combined findings of failed aggregation on the ristocetin test with large platelets on peripheral blood smear favored the diagnosis of BSS. Flow cytometry was advised but was not conducted due to financial and resource constraints.

After confirming the patient's diagnosis, her family was counseled about a supportive management plan that included folic acid, multivitamins, and desmopressin. For active bleeding episodes, tranexamic acid–soaked gauzes were provided for home treatment. Uncontrolled bleeding episodes were treated with intravenous tranexamic acid and platelet transfusions dosed according to the severity of the bleeding. The brother was managed with the same regimen mentioned above.

## 3 DISCUSSION

BSS is a rare inherited platelet dysfunctional disorder characterized by giant platelets and bleeding episodes manifesting as clinical symptoms such as epistaxis, menorrhagia, and easy bruising.^9^ A study in a tertiary care hospital in Pakistan reported having around 49 patients with BSS in 7 years, revealing the increasing need to raise awareness among the general public.^9^ As this disorder presents most commonly as a recessive trait, consanguinity must be assessed, which is reasonably prevalent in Pakistan, with 50% of cases derived from consanguineous marriages.^10^ The largely autosomal nature of the disease means that both men and women are affected equally, as our patient and her brother both have the condition.

The complexity of the genetics of BSS can be seen in its pathophysiology. The genetic mutations create an absence of a major carbohydrate‐containing protein complex on platelet surfaces, which results in a severe deficiency of four glycoproteins: GPIbα, GPIbβ, GPIX, and GPV.^11^ These mutations can be monoallelic and biallelic, resulting in quantitative defects and/or a complete or partial malfunctioning of the von Willebrand factor receptor. In its recessive form, the syndrome encompasses giant platelets with bleeding tendencies, as seen in our patient; and in its dominant form, there is only moderate thrombocytopenia with bleeding tendencies.^11^ Apart from the symptomatology, current preventive and supportive management remains the same for affected individuals regardless of their allelic variant.

Although laboratory investigations confirm the diagnosis of BSS, these findings are likely similar to those of other platelet disorders. Platelet aggregation studies (i.e., light transmission aggregometry) showing decreased or absent response to ristocetin indicate BSS, which is further confirmed by flow cytometry depicting the low expression of Glycoprotein Ib.^12^ Diagnosis can also help in differentiating between immune thrombocytopenic purpura (ITP) and von Willebrand Disease (vWD) because of common symptomatology. In the case of BSS, on platelet aggregation studies, there is decreased or absent response to ristocetin that cannot be corrected by adding normal plasma compared to vWD, which shows a strong response to the addition.^12^ BSS is more likely to be seen in consanguineous marriages due to its autosomal recessive inheritance. In contrast, in the case of ITP, an acquired disease, the presence of a family history of ITP can confirm its diagnosis. Moreover, standard lab investigations for BSS can successfully prevent misdiagnosing BSS as ITP.^13^ Due to the extensive overlapping of clinical features, BSS patients started on therapies for ITP and vWD show little to no benefit. Therefore, accurate diagnosis is necessary to ensure proper management of the affected individual. In our case, a ristocetin test and a peripheral blood smear confirmed the diagnosis of BSS.

In managing BSS, we must first address preventive care. Patients with BSS can live normally if proper education and primary health care are provided. First, thorough education should be provided for the patient and their family to highlight the bleeding risks and preventive measures to avoid such episodes (e.g., safe play, avoiding high‐risk sports, and applying pressure on wounds). In addition, knowing which medications to avoid that can trigger bleeding episodes or hinder clot formation (e.g., heparin, warfarin, aspirin, ibuprofen, and naproxen) is also necessary. Parents in consanguineous marriages and those who are carriers are at risk for their fetuses to acquire BSS. Therefore, proper counseling should be done for these families antenatally. Mothers with BSS may develop severe hemorrhages and should be monitored accordingly throughout pregnancy and postpartum for any bleeding symptoms. If recombinant factor VII has been given, thrombo‐prophylaxis should be considered individually.^14^ During labor, neuraxial anesthesia should be avoided because of the risk of hemodynamic instability in the patient. However, when required, uterotonics, HLA‐matched platelets, tranexamic acid, and rfVIIa should be used.^12^ Neonates born to mothers with BSS must also be monitored closely as they may sometimes require multiple platelet transfusions throughout their lives. For BSS patients, the United States Department of Agriculture recommends a diet rich in whole grains, fruits, and vegetables and low in solid fats, added sugars, and salt.^12^ BSS patients should also be informed about crossmatching and blood typing ahead of time in case any transfusions are needed under emergent conditions. However, the patient and the family members were not counseled before the current interaction. This case represents a significant problem in Pakistan, where patients know very little about their condition, which leads to many preventable complications of illness. Educating patients will prepare them to handle their situation better and give them the confidence to manage any associated psychosocial aspects.

The first pharmacological treatment for bleeding episodes in BSS is platelet transfusions, but caution is mandated, as BSS patients can develop antibodies against missing glycoproteins.^12^ For mucocutaneous bleeding and menorrhagia episodes, tranexamic acid can be used along with local efforts such as nasal packing, compression sponges, and hormonal treatments.^12^ In our patient, tranexamic acid is usually helpful in minor bleeding episodes; however, platelet transfusions were required for this episode, as the bleeding was not resolved with tranexamic acid alone. Special care must be taken when managing patients with comorbidities such as pulmonary hemorrhages, as treating such patients with fibrinolytic can cause respiratory or renal failure.^12^ Furthermore, despite desmopressin releasing vWF from platelets, it has limited utility in managing BSS due to the defective GPIB‐IX‐V complex.^12^ Other treatment modalities such as eltrombopag, recombinant factor VII, allogeneic stem‐cell transplantation, and gene therapy are still under investigation. Special consideration should be given to pregnant patients with BSS.

## 4 CONCLUSIONS

As there are limited reports of BSS in the literature due to its rare presentation, clinicians must practice increased vigilance when presented with a patient with bleeding disorder symptoms. In societies where consanguineous marriages are common, preconception counseling can be vital in raising awareness of BSS. Timely diagnosis can prevent further complications and vastly improve the prognosis of the disease. The battle against BSS requires a multidisciplinary team approach involving physicians of different specialties and dieticians, public health specialists, and therapists so that optimal management plans tailored to the populations' physical, psychological, and societal needs are provided.

## AUTHOR CONTRIBUTIONS

**Iqra Effendi:** Conceptualization; data curation; writing – original draft; writing – review and editing. **Ahsan Nadeem:** Conceptualization; data curation; writing – original draft; writing – review and editing. **Sara Sarfraz:** Conceptualization; data curation; writing – original draft; writing – review and editing. **Mubasshar Shahid:** Conceptualization; data curation; writing – original draft; writing – review and editing. **Minaam Farooq:** Conceptualization; data curation; writing – original draft; writing – review and editing. **Ayush Anand:** Conceptualization; supervision; validation; visualization; writing – review and editing.

## FUNDING INFORMATION

The authors did not receive any funding for this manuscript.

## CONFLICT OF INTEREST STATEMENT

The author(s) have no conflict of interests to declare.

## ETHICS STATEMENT

Our institution does not require ethical approval for reporting individual cases or case series.

## CONSENT

Written informed consent was obtained from the patient(s) for their anonymized information to be published in this article.

## Data Availability

All data pertaining to this case is available within this manuscript.
